# Prevention of Cyclophosphamide-induced Accelerated Diabetes in the NOD Mouse by Nicotinamide or a Soy Protein-based Infant Formula

**DOI:** 10.1155/EDR.2000.299

**Published:** 2000

**Authors:** S. Reddy, M. Karanam, E. Robinson

**Affiliations:** 1 Department of Paediatrics Faculty of Medicine and Health Science University of Auckland Private Bag Auckland 92019 New Zealand; 2 Zhe Research Centre for Developmental Medicine and Biology Faculty of Medicine and Health Science University of Auckland Private Bag Auckland 92019 New Zealand; 3 Department of Community Medicine Faculty of Medicine and Health Science University of Auckland Private Bag Auckland 92019 New Zealand

## Abstract

Spontaneous diabetes in the NOD mouse can be prevented by nicotinamide or by an infant formula diet in which the protein source is replaced with casein hydrolysate (Pregestimil) or soy protein (Prosobee). NOD mice maintained on the standard diet (chow and water) and given cyclophosphamide (Cy) at day 95 develop accelerated and synchronised diabetes within 14 days. Here, we compared the ability of oral nicotinamide or Prosobee, either given alone or concurrently, from weaning, in preventing diabetes in the Cy model. The resulting insulitis and the expression of intra-islet inducible nitric oxide synthase (iNOS) were examined at days 0, 4, 7, 11 and 14 following Cy administration. Intra-islet CD4 and CD8 cells and macrophages were also enumerated at day 11. In mice given the standard diet and injected with Cy at day 95 (group 5), diabetes developed in 7/11 mice, 14 days later. Mice exposed to oral nicotinamide (group 2), Prosobee (group 3) or both (group 4), did not develop the disease during this period and until a further 30 days (*p* =  0.03). In mice exposed to the standard diet and without Cy treatment (group 1) the insulitis scores increased slowly until day 11 and then declined slightly at day 14 whereas mice exposed to the same diet but given Cy at day 95, showed a sharp decline at day 4 followed by a rapid increase between day 7–14. However, in mice given either nicotinamide, Prosobee or both, the insulitis scores at most time-points were generally lower than in Cy-teated animals on the standard diet. In the latter group, CD4 and CD8 cells and macrophages were also higher at day 11 than all other 4 groups (CD4: *p* < 0.05; CD8: *p* < 0.05; macrophages: *p* < 0.0001). The number of iNOS labelled cells increased progressively in mice on the standard diet and given Cy and were significantly higher at days 4, 7 and 11 than in the 3 dietary groups. Thus, oral nicotinamide or Prosobee, either alone or together, prevents Cy induced diabetes in the NOD mouse. The protective diets suppress Cyinduced intra-islet immune cell influx and iNOS expression.


*Int. Jnl. Experimental Diab. Res.*, Vol. 1, pp. 299–313
Reprints available directly from the publisher
Photocopying permitted by license only

© 2001 OPA (Overseas Publishers Association) N.V.
Published by license under
the Harwood Academic Publishers imprint,
part of The Gordon and Breach Publishing Group.
Printed in Malaysia.

### Prevention of Cyclophosphamide-induced Accelerated Diabetes in the NOD Mouse by Nicotinamide or a Soy Protein-based Infant Formula

S. REDDY[a,b,*], M. KARANAM[a] and E. ROBINSON[c]

[a]*Department of Paediatrics,* [b]*The Research Centre for Developmental Medicine and Biology,* [c]*Department of Community Medicine, Faculty of Medicine and Health Science, University of Auckland, Private Bag 92019, Auckland, New Zealand*

*(Received 15 May 2000; In final form 24 August 2000)*

**Spontaneous diabetes in the NOD mouse can be prevented by nicotinamide or by an infant formula diet in which the protein source is replaced with casein hydrolysate (Pregestimil) or soy protein (Prosobee). NOD mice maintained on the standard diet (chow and water) and given cyclophosphamide (Cy) at day 95 develop accelerated and synchronised diabetes within 14 days. Here, we compared the ability of oral nicotinamide or Prosobee, either given alone or concurrently, from weaning, in preventing diabetes in the Cy model. The resulting insulitis and the expression of intra-islet inducible nitric oxide synthase (iNOS) were examined at days 0, 4, 7, 11 and 14 following Cy administration. Intra-islet CD4 and CD8 cells and macrophages were also enumerated at day 11. In mice given the standard diet and injected with Cy at day 95 (group 5), diabetes developed in 7/11 mice, 14 days later. Mice exposed to oral nicotinamide (group 2), Prosobee (group 3) or both (group 4), did not develop the disease during this period and until a further 30 days ($p = 0.03$). In mice exposed to the standard diet and without Cy treatment (group 1) the insulitis scores increased slowly until day 11 and then declined slightly at day 14 whereas mice exposed to the same diet but given Cy at day 95, showed a sharp decline at day 4 followed by a rapid increase between day 7–14. However, in mice given either nicotinamide, Prosobee or both, the insulitis scores at most time-points were generally lower than in Cy-treated animals on the standard diet. In the latter group, CD4 and CD8 cells and macrophages were also higher at day 11 than all other 4 groups (CD4: $p < 0.05$; CD8: $p < 0.05$; macrophages: $p < 0.0001$). The number of iNOS labelled cells increased progressively in mice on the standard diet and given Cy and were significantly higher at days 4, 7 and 11 than in the 3 dietary groups. Thus, oral nicotinamide or Prosobee, either alone or together, prevents Cy induced diabetes in the NOD mouse. The protective diets suppress Cy-induced intra-islet immune cell influx and iNOS expression.**

*Keywords*: NOD mice; Cyclophosphamide; Nicotinamide; Prosobee; Diet; Prevention; Nitric oxide

### INTRODUCTION

Several studies have described successful prevention of insulin-dependent diabetes mellitus (IDDM) in the NOD mouse. Some of these are

*Corresponding author. Tel.: 64-9-3737599, Fax: 64-9-3737486, e-mail: s.reddy@auckland.ac.nz currently being evaluated in human clinical trials.[1] Nicotinamide injected into adult NOD mice over a protracted period or given orally as drinking water from weaning prevents diabetes.[2,3] Complete protection is also achieved with the complete infant formula, Pregestimil or Prosobee in which the protein source is bovine casein hydrolysate or soy protein, respectively, when introduced from weaning.[4,5] Epidemiological reports suggest that ingestion of bovine milk as the sole nutritional source by the human newborn and during infancy may be an important etiological factor for the development of IDDM.[6,7] These studies have been supported by the recent demonstration of increased cellular reactivity of blood mononuclear cells to bovine $\beta$-casein, lactoglobulin and humoral reactivity to bovine serum albumin (BSA) and $\beta$-lactoglobulin, in newly-diagnosed diabetic subjects.[8-10] However, the role of cow's milk components in the etiopathogenesis of human IDDM has recently been questioned.[11-14] A large clinical trial aimed at testing the efficacy of bovine casein-hydrolysate based . infant formula in preventing or delaying diabetes in high risk newborns is in progress.[15]

Oral nicotinamide also delays the onset of IDDM in diabetes-prone children.[16,17] A large case-controlled clinical trial involving nicotinamide prophylaxis is in progress (the European Nicotinamide Diabetes Intervention Trial (END-IT)). However, the efficacy of nicotinamide in preventing IDDM in high risk individuals and with an assumed rapid progression to disease may be low, as concluded from the recent Deutsche Nicotinamide Intervention Study (DENIS).[18]

Cyclophosphamide (Cy) when given to NOD mice as a single injection promotes a rapid influx of immune cells into the islets resulting in accelerated and synchronised diabetes.[19,20] In this intensified model, nitric oxide (NO) may be a key molecular mediator of beta cell destruction since the heightened insulitis observed immediately preceding onset of diabetes is associated with significant up-regulation of intra-islet inducible nitric oxide synthase (iNOS).[20,21] Cyclophosphamide induced diabetes in the NOD mouse may have some immunopathogenetic similarities to the rapidly progressing form of human IDDM. However, it is unclear if nicotinamide and the bovine casein-free infant formula, previously shown to protect NOD mice from spontaneous diabetes, are equally effective in preventing diabetes in this accelerated model. Here, we have, therefore, tested whether oral nicotinamide or Prosobee, introduced to female NOD mice from weaning, either alone or in combination, would successfully prevent Cy-induced diabetes. We have also examined some of the likely immune and molecular mechanisms which may underlie any protective effects associated with such dietary treatments, including their effects on insulitis, intra-islet immune cell numbers and the expression of iNOS.

### MATERIALS AND METHODS

### Animals

A colony of NOD mice was established in Auckland, New Zealand, under conventional conditions, from three breeding pairs originally obtained from the University of Ehime, Japan. The rate of spontaneous diabetes among the Auckland colony is 50% among females and $< 1\%$ among males at 120 to 250 days. Diabetes was defined as the presence of heavy glucosuria on three consecutive days detected by Testape (Eli Lilly, St Louis, IN, USA) and confirmed by a hyperglycaemic value of $> 12$ mM in blood samples from the tail (Medisense blood glucose meter).

### Study Groups

Female NOD mice were generated from 10 breeding pairs through repeated breeding, weaned at day 21 and allocated at random into

5 groups (23 mice per group). They were exposed to various diets from day 21 until day 250 for group 1 mice and until day 140 for mice from groups 2–5. The 5 experimental groups were as follows:

Group 1: standard mouse chow (Diet 86, see below) + drinking water.
Group 2: standard mouse chow + 1% nicotinamide (New Zealand Tablet Manufactures) as drinking water.
Group 3: Prosobee infant formula (Mead Johnson and Co, Evansville, IN, USA, see below for composition) + drinking water.
Group 4: Prosobee infant formula + 1% nicotinamide as drinking water.
Group 5: standard mouse chow + drinking water.

The composition of standard mouse chow (Diet 86) and Prosobee infant formula was identical to those used in our previous studies in the NOD mouse.[5]

### Chow (Diet 86)

Diet 86 contains wheat, barley, meat and bone meal (derived from cows, pig and sheep and includes offal) and is also supplemented with minerals, trace elements and vitamins.

### Prosobee Powder

Ingredients: Corn syrup solids, soy protein isolate, corn oil, monoglyceride, minerals (potassium citrate, calcium phosphate, calcium chloride, magnesium chloride, calcium hydroxide, potassium bicarbonate, sodium citrate, potassium hydroxide, ferrous sulphate, zinc sulphate, manganese sulphate, cupric sulphate and sodium iodide), vitamins (vitamin A, palmitate, calciferol, DL-$\alpha$-tocopherol acetate, sodium ascorbate, folic acid, thiamine hydrochloride, riboflavin, niacinamide, pyridoxine hydrochloride, cyanocobalamin, biotin, calcium pantothenate and phytonadione), choline bitartrate, L-methionine, taurine and ascorbyl palmitate.

The principal constituents of Prosobee and chow are given below:

| Composition | Prosobee | Chow |
|---|---|---|
| Protein g | 15.8 | 18.1 |
| Fat g | 27.9 | 5.6 |
| Carbohydrate g | 51.4 | 57 |
| Minerals g | 2.6 | – |
| Water g | 2.5 | 9.74 |
| Choline mg | 66 | 30 |
| Calcium g | 0.427 | 1.5 |
| Salt g | 1.03 | 1.36 |
| Amino acids | – | 6.0 |
| Phosphorus g | 0.310 | 0.84 |
| Iodine μg | 35 | 70 |
| Linoleic acid g | 8.0 | 1.27 |
| Xanthophyll mg | – | 8 |

### Administration of Cyclophosphamide and Tissue Collection

Cy was administered to NOD mice as described recently.[20] At day 95, all except 3 mice from groups 2–5 were injected with Cy (Sigma, St. Louis, USA) at a dose of 300 mg/kg body weight by the intra-peritoneal route.[20] Group 1 mice (controls) received diluent (sterile water). Following injection of Cy or diluent, 3 mice from each group were sacrificed at days 0 (day 95 and without Cy administration), 4, 7 and 11 and the pancreas harvested (see below). The remaining 11 mice from each group were followed until day 14 for development of diabetes. At day 14, three mice from groups 1–4 and all 7 diabetic mice from group 5 were killed and the pancreas removed. The remaining non-diabetic mice from the day 14 group were monitored for diabetes for a further 30 days (groups 2–4) or until day 250 (group 1, spontaneous diabetes group).

At the various time-points described above animals were killed by cervical dislocation and almost the entire pancreas with a small portion of the adjacent spleen, was removed, embedded in OCT and snap-frozen in isopentane cooled in liquid nitrogen. Tissues were stored at −70°C until histochemical and immunohistochemical analysis.

### Primary Antibodies, Non-immune IgG and Normal Sera

Polyclonal antibodies to mouse macrophage iNOS (rabbit IgG fraction, 1 mg/ml) were prepared by Dr. C. Nathan, Cornell University Medical College, New York, USA and supplied by Upstate Biotechnology, New York, USA. The monospecificity of this antibody for mouse iNOS has been reported previously, and it has been employed for immunohistochemical labelling of the enzyme in lipopolysaccharide (LPS) and interferon-$\gamma$ (IFN-$\gamma$) activated mouse peritoneal macrophages.[22] This antibody has subsequently been employed by the same authors for immunolocalization of iNOS in pancreatic sections of the BB rat and by us and other investigators in the NOD mouse.[20,23,24]

Dr. H. Georgiou of the Walter and Eliza Hall Institute, Melbourne, Australia supplied rat monoclonal antibodies to mouse macrophages (MAC-1, clone M/170), CD4 (clone GK 1.5) and CD8 (clone 53-6.72) in the form of culture supernatants. The same monoclonal antibodies have been previously used for immunohistochemical staining of sections of pancreas from the NOD mouse in this laboratory.[25,26]

In the immunohistochemical procedure, all primary antisera were titrated to give maximal immunohistochemical reactivity. Normal sera from the goat, sheep, donkey, rabbit, guinea pig, rat and mouse were available in this laboratory.

### Histochemical Staining and Evaluation of Insulitis

Serial frozen sections (8 μm) were prepared from different levels of the pancreas, thaw-mounted on glass slides and fixed in cold acetone for 10 min and stored at −20°C until required. Sections from each level were stained by Haematoxylin and Eosin (H and E) and the islets graded for the severity of insulitis from a scale of 0−4 as reported previously.[20] By this method, islets devoid of any mononuclear cells = 0, minimum focal islet infiltrate = 1+; peri-islet infiltrate of < 25% of islet circumference = 2+; peri-islet infiltration and < 50% intra-islet infiltrate = 3+; intra-islet infiltration > 50% of islet area = 4+. All slides were coded and at least 10 separate islets from different levels of the pancreas of each animal were scored. The insulitis score (%) for each study group was calculated as follows:

Sum of (1 × number of islets with 1+, 2 × number of islets with 2+, 3 × number of islets with 3+, 4 × number of islets with 4+) divided by 4 × total number of islets scored. The ratio obtained was expressed as a percentage. The insulitis score (%) for each study group was expressed as the mean ± SEM.

### Immunolabelling and Enumeration of CD4 and CD8 Cells and Macrophages

Pancreatic sections at day 11 belonging to the 5 groups were immunolabelled by the immunoperoxidase technique for CD4 and CD8 cells and macrophages as described previously, with minor modifications.[3,26] During the immunohistochemical procedure (described below) sections were washed in excess phosphate-buffered saline (PBS), pH 7.5, before addition of each immunological reagent. Prior to commencement of immunohistochemical labelling, sections were routinely re-fixed briefly in cold acetone and equilibrated in PBS.

Following blocking with 5% normal rabbit serum and 1% rabbit anti-mouse IgG (Sigma, St. Louis, MO, USA), monoclonal antibodies to CD4 and CD8 cells and macrophages were applied to serial sections of pancreas and incubated for 2.5 h at 37°C. After washing, sections were incubated with rabbit anti-rat IgG-biotin (1 : 100, Vector, Burlingame, CA, USA) for 1 h at 37°C. Endogenous peroxidase activity was blocked with 0.5% $H_2O_2$ in methanol. Sections were washed and reacted with Vectastain Elite ABC-peroxidase reagent (Vector) for 1 h at 37°C. Sections were exposed to diaminobenzidine hydrochloride in the presence of $H_2O_2$ to reveal the presence of immunoreactive cells and counterstained with haematoxylin. They were dehydrated with graded series of alcohol, cleared in xylene and viewed with a Leica microscope attached to a video monitor (see below). Macrophage immunolabelling was also carried out by immunofluorescence as described previously.[24] Following incubation with Mac-1 antibody, sections were reacted with rabbit anti-rat IgG-biotin followed by streptavidin-Texas Red (Jackson Immunoresearch Laboratories, West Grove, PA, USA). Sections were prepared for microscopic examination.

The number of immunoperoxidase-stained CD4 cells, CD8 cells or macrophages present within the islets and immediately outside but in close apposition to the islet boundary were either counted microscopically or following display on a monitor.[20, 26] At least 10 islets from each animal were analysed. Results were expressed as the mean number of immune cells per islet for each group ± SEM.

### Immunolabelling and Enumeration of iNOS

Immunohistochemical labelling of iNOS was carried out as described recently, with minor modifications.[20, 24] Following blocking with normal sheep serum, sections were incubated with anti-iNOS (1 : 200) for 16 h at room temperature followed by incubation with goat anti-rabbit IgG-biotin (1 : 200, Jackson Immunoresearch Laboratories) for 1 h at 37°C. Sections were finally incubated with streptavidin-Texas Red (1 : 200, Jackson Immunoresearch Laboratories) for 1 h at 37°C, washed and mounted with glycerol : PBS before microscopic examination and photomicrography.

Adjacent sections from day 11 pancreas were also immunolabelled for macrophages as described above. Macrophage-positive cells were visualized using streptavidin-Texas Red instead of streptavidin-peroxidase.

The number of intra-islet iNOS positive cells were enumerated at days 0, 4, 7 and 11 (days 0, 4 and 7 for group 5) either by direct microscopic visualization or following photography and were expressed as the mean ± SEM per islet.

### Light Microscopy and Photography

Sections were examined with a Leica light or Olympus UV-visible microscope equipped with excitation filters for Texas Red (568 nm) and fluorescein (488 nm) and mercury and halogen lamps. At least 10 islets from each pancreas and the entire exocrine region on each slide were analyzed.

Appropriate fields from light immunofluorescent and immunoperoxidase stained sections and following H and E staining were photographed with a Kodak Ektachrome 200 ASA film and the images scanned to a photo compact disk.

### Immunohistochemical Controls

In the immunohistochemical procedure for iNOS, the primary antibody was replaced with buffer, normal rabbit IgG or normal sera from a rabbit, mouse, rat or guinea pig at equivalent dilutions. In addition, primary and secondary steps were omitted.

### Statistical Analysis

A Chi square test was used to investigate differences in diabetes rate among the groups. An exact probability was calculated because of the small expected numbers. An analysis of variance was used to investigate differences in insulitis scores in the 5 groups and the differences over time. An arc sine transformation was used to allow for the distribution of the insulitis scores. The level of significance was found using Scheffes test. Intra-islet CD4 and CD8 cells and macrophages were analysed separately by analysis of variance. Levels of significance were determined by Scheffes test. For intra-islet iNOS cell numbers, a logistic regression was used to investigate whether there was a difference between the groups and a change over time. A Chi square test was employed to determine significance between time and group.

### RESULTS

### Incidence of Diabetes

The incidence of Cy-induced diabetes in the 5 experimental groups is shown in the following table:

| Groups | Cy-induced diabetes |
|---|---|
| 1: Standard diet and without Cy | 0/11 |
| 2. Standard diet and nicotinamide + Cy | 0/11 |
| 3. Prosobee + Cy | 0/11 |
| 4. Prosobee and nicotinamide + Cy | 0/11 |
| 5. Standard diet + Cy | 7/11 |

In group 5, Cy administration resulted in diabetes in 7/11 mice (63%) 14 days later. During the same period, none of the 11 mice from groups 1–4 developed the disease ($p = 0.03$, Chi square test). In groups 2–4, none of the 8 remaining mice developed diabetes when followed for 45 days after Cy administration. In mice which did not receive Cy (group 1, chow and water, $n = 8$) spontaneous diabetes developed in 4/8 mice (1 at day 128, 2 at day 142 and 1 at day 182). There were no differences in body weights between the various diet- and Cy-treated groups.

### Severity of Insulitis

Figure 1 shows representative islets from the 5 groups following H and E staining of pancreatic sections. In group 1, significant infiltration was seen from day 0 to day 14 (Fig. 1a, b). In group 2 (nicotinamide treated), majority of the islets were either insulitis free or had peri-islet infiltration (Fig. 1 c–h). A similar pattern was also seen in group 3 (Prosobee-fed) until day 7 (Fig. 1 i, j). However, the severity of insulitis became more pronounced by days 11 and 14 (Fig. 1 k, l). In group 4 (Prosobee + nicotinamide), many islets were well preserved throughout the 14 day study period, although some were heavily infiltrated (Fig. 1 m–o). In group 5 (Cy treated, chow and water), islets became heavily infiltrated from day 7 onwards (Fig. 1 p–r).

The mean ± SEM insulitis scores (%) in the various experimental groups at days 0, 4, 7, 11 and 14 are shown in Figure 2. For group 5, the insulitis score at day 14 represents the mean of 3 diabetic mice whereas for groups 1–4, the means are from non-diabetic mice (3 mice per group).

In mice exposed to the standard diet and given Cy, the insulitis score declined rapidly at day 4 but increased sharply by day 7 and remained elevated at days 11 and 14 (Fig. 2). Although the insulitis scores increased at days 11–14, diabetes was not observed for an additional month. The reasons for the continued protection are unclear but may be due to a switch towards protective cytokines in the islet infiltrate. In all 5 groups, there was a significant stage effect ($F_{5.71} = 6.47$; $p = 0.0001$) with the insulitis scores generally increasing over time. There were differences in the level of insulitis in the 5 groups as a whole ($F_{4.754}$; $p < 0.001$). A multiple comparison of the means using Scheffes test showed that the insulitis scores were significantly higher in group 5 than in groups 2–4 ($p = 0.05$). They were also higher in group 1 than in groups 2–4 ($p = 0.05$).

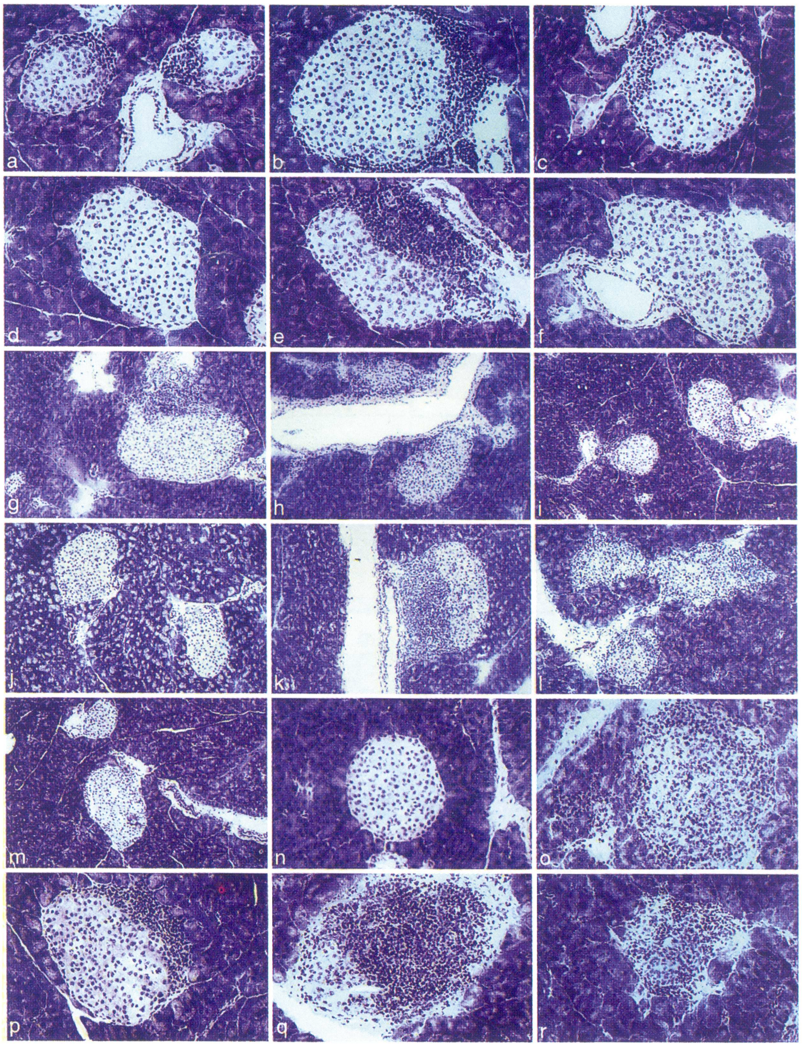

FIGURE 1 Photomicrographs of pancreatic sections stained by H and E, showing representative islets with insulitis from groups 1–5. a, b: Group 1, (a): day 4 and (b): day 7. c–h: Group 2, (c,d): day 0; (e,f): day 4; (g): day 7; (h): day 14. Note well-preserved islets at most stages. i–l: Group 3, (i): day 0; (j): day 7; (k): day 11; (l): day 14. Note advanced insulitis at days 11 and 14. m–o: Group 4, (m): day 0; (n): day 7; (o): day 11. Note insulitis-free islets at days 0 and 7 but advanced insulitis at day 11. p–r: Group 5, (p): day 0; (q): day 7; (r): day 11. Note advanced insulitis from day 7. (a–f) and (n–r): × 200; (g–m): × 100. (See Color Plate II).

### Intra-islet CD4 and CD8 T Cells and Macrophages

In all 5 experimental groups, the intra-islet distribution of CD4 and CD8 cells and macrophages at day 11 was variable. This is shown in Figure 3.

Figure 4 shows the mean ± SEM number of CD4 and CD8 cells and macrophages per islet at day 11 in the 5 groups. All 3 cell-types showed differences between the groups (macrophages: $F_{4.169} = 19.44$, $p < 0.0001$; CD4: $F_{4,170} = 4.22$, $p < 0.003$; CD8: $F_{4.169} = 13.89$, $p < 0.0001$). Intra-islet macrophage numbers were significantly higher in group 5 than in the other 4 groups ($p < 0.05$). They were also lower in the control group (group 1) than in groups 2 and 4

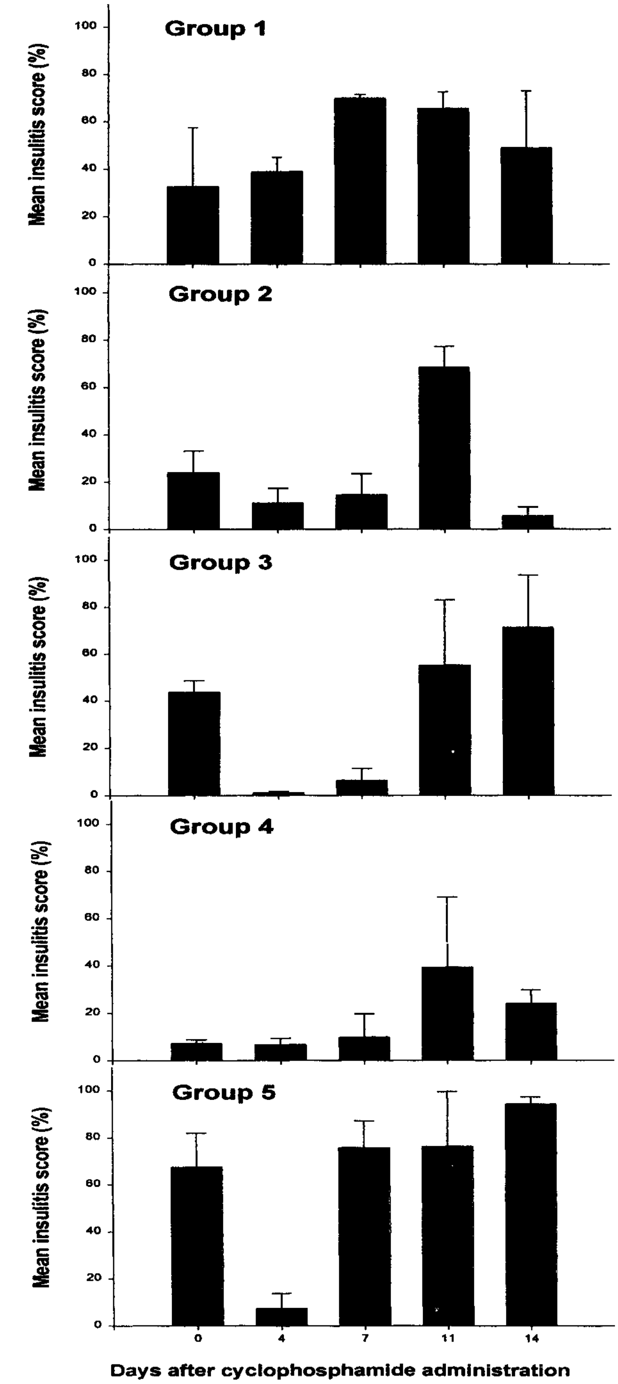

FIGURE 2 The mean insulitis scores (% ± SEM) at days 0, 4, 7, 11 and 14 in cyclophosphamide-treated (groups 2–5) or diluent-treated (group 1) mice. Insulitis scores were obtained from 3 mice at each time-point. For group 5, the insulitis at day 14 represents the mean from 3 diabetic mice whereas for groups 1–4, the scores are from 3 non-diabetic mice per group.

($p < 0.05$). No significant differences in macrophage numbers were observed between the 3 dietary groups (groups 2–4).

Intra-islet CD4 cells were significantly higher in group 5 than in groups 3 and 4 ($p < 0.05$), whereas intra-islet CD8 cells were significantly higher in group 5 than in groups 1–4 ($p < 0.05$).

### Intra-islet iNOS Expression

The distribution of intra-islet iNOS immunoreactive cells in the various study groups is shown in Figure 5. In group 1, expression of the enzyme was observed rarely and only in a few infiltrated islets (Fig. 5 a,b). Weak intra-islet expression for the enzyme was first observed at day 4 only in group 5 mice and became more numerous and intense at days 7 and 11. In groups 2–4, iNOS was expressed in a small number of intra-islet cells at days 7 and 11. In groups 1–4, several islets showed an absence or only minimal expression of iNOS despite significant macrophage infiltration (Fig. 5).

The mean ± SEM number of iNOS expressing cells per islet in the various experimental groups were analysed at days 0, 4, 7 and 11 and are shown in Figure 6. In group 5, a large proportion of islets at day 11 showed extremely intense staining for the enzyme, and thus did not permit reliable enumeration. Therefore, iNOS positive cells in this group were enumerated at days 0, 4, and 7 only. As seen in Figure 6, iNOS expressing cells were first observed among group 5 mice at day 4, which then increased sharply at days 7 and 11. The enzyme was observed in only a few islet cells in mice from group 1.

The mean number of intra-islet iNOS expressing cells was higher in group 5 at days 4, 7 and 11 ($p = 0.001$, Chi square) compared with comparable time-points from groups 1–4. The number of iNOS expressing cells at day 7 was not significantly different between groups 2–4. At day 11, group 2 mice had slightly higher iNOS expressing cells compared with groups 3 and 4.

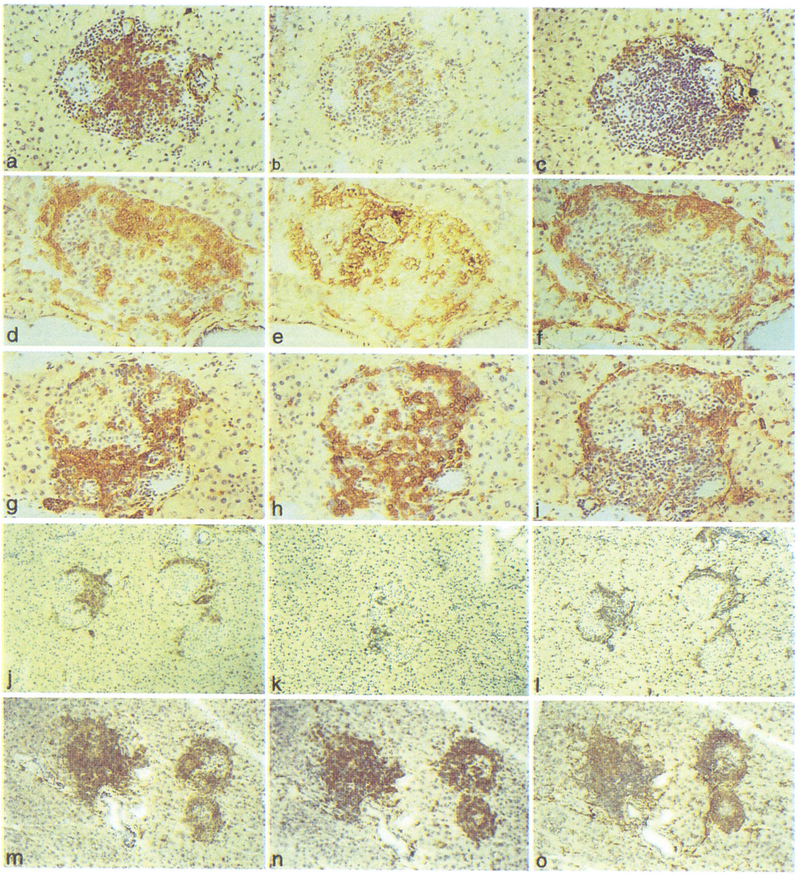

FIGURE 3 Representative photomicrographs of pancreatic islets from day 11 stained by immunoperoxidase for CD4 and CD8 cells and macrophages, respectively, in groups 1 (a – c), 2 (d – f ), 3 ( g – i), 4 (j – l) and 5 (m – o). In groups 1, 2, 3 and 5, the same islets are shown from 3 serial sections whereas in group 4, (j) and (l) show the same islets in adjacent sections. (a – i): × 200; (j – o): × 100. (See Color Plate III).

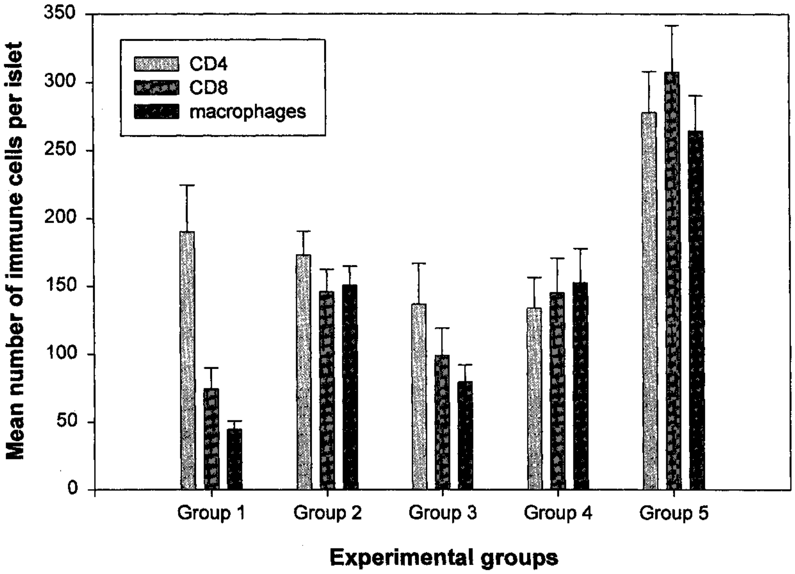

FIGURE 4 The mean ± SEM number of CD4 cells, CD8 cells and macrophages per islet at day 11 in groups 1 – 5.

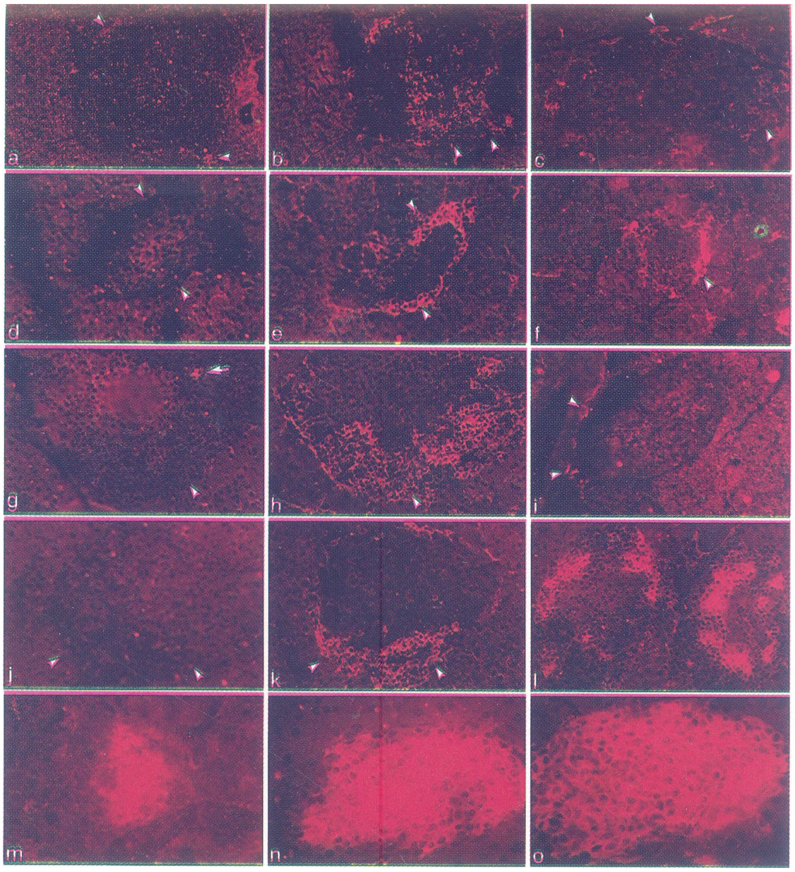

FIGURE 5 Immunofluorescence photomicrographs showing pancreatic islets at day 11 from groups 1–5, immunolabelled for iNOS and macrophages.
a,b: Group 1. (a) and (b): two serial sections showing the same islet immunolabelled for iNOS (a) and macrophages (b). Arrowheads point to only a few iNOS cells (a) and to numerous macrophages (b).
c–e: Group 2: (c): iNOS (arrowheads). (d) and (e) are two serial sections showing the same islet immunolabelled for iNOS (d) and macrophages (e). Arrowheads point to numerous macrophages distributed as a peri-islet band while arrowheads in (d) point to the corresponding area which is negative for iNOS.
f–h: Group 3: (f): iNOS (arrowhead). (g) and (h): same islet from 2 serial sections immunolabelled for iNOS (g) and macrophages (h). Arrowhead in (h) points to numerous intra-islet macrophages while arrowheads in (g) point to the corresponding area which is negative for iNOS. Only a few iNOS positive cells are visible (g) (arrow).
i–k: Group 4: (i): iNOS (arrowheads). (j) and (k) show the same islet from 2 serial sections immunolabelled for iNOS (j) and macrophages (k). Arrowheads in (k) point to numerous intra-islet macrophages while arrowheads in (j) point to the corresponding area, which is negative for iNOS.
l–o: Group 5: (l–n) show islets strongly immunolabelled for iNOS. (l) shows 2 adjacent islets with iNOS immunolabelling in selective intra-islet cells. (m) and (n): islets with predominant and intense iNOS immunolabelling. (o) is a serial section of (n) showing the same is let stained for macrophages. (a–l): × 200; (m–o): × 400. (See Color Plate IV).

### Control Results for Immunohistochemistry

There was an absence of immunolabelling for iNOS when the primary immune IgG was replaced by normal rabbit serum or normal rabbit IgG at equivalent dilution or by PBS. Labelling was also absent when anti-iNOS and biotinylated goat anti-rabbit IgG were omitted, or when the secondary step was replaced with biotinylated antibodies against rat IgG. In the immunohistochemical procedure for CD4 and CD8 cells and macrophages there was an

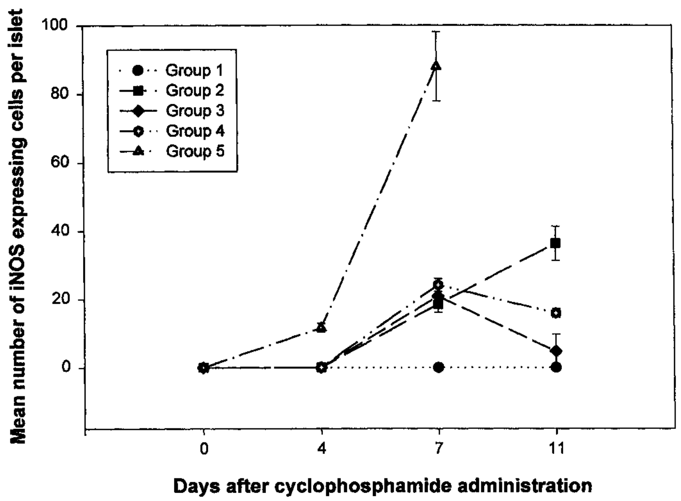

FIGURE 6 Mean number ± SEM of iNOS expressing cells per islet at days 0, 4, 7 and 11 (groups 1–4) and days 0, 4 and 7 (group 5). The mean number of iNOS positive cells per islet at day 11 in group 5 far exceeded other time-points (see Fig. 6 (m–o)).

absence of labelling when the primary antibodies were replaced with normal rat serum (diluted 1:50 and 1:100). Immune cell staining was not observed when the secondary step was excluded.

### DISCUSSION

In this study we have employed the Cy model of accelerated diabetes in the NOD mouse to test the efficacy of oral nicotinamide and Prosobee, either given alone, or in combination, in preventing diabetes. The three dietary treatments were shown to successfully prevent diabetes in the Cy model. The same dietary approaches have been shown previously to produce long-lasting protection from spontaneous diabetes in the NOD mouse.[5] In the present study, we also examined some of the likely immune mechanisms underlying dietary protection in the Cy model.

NOD mice maintained on the standard diet (chow and water) and given a single injection of Cy at day 95 developed diabetes at a rate of 63% in a synchronous manner 14 days later. However, prior exposure to oral nicotinamide or Prosobee, either alone or together from weaning, resulted in complete protection from the more intensified form of diabetes induced by Cy. Other dietary ploys, such as Pregestimil or Nutramigen, a bovine casein-hydrolysate based infant formula, may also be equally protective in mice given Cy, since the same formulae prevent spontaneous disease in the NOD mouse.[4,5,27,28]

In the NOD mouse, islet cell directed autoantibodies and mononuclear cell infiltration of pancreatic islets are detectable after weaning.[29,30] Insulitis increases progressively soon after and by day 90–100 many islets have severe insulitis.[3,26,30] Cy given to NOD mice maintained on the standard diet induces a massive and progressive infiltration of immune cells within the islets, as shown in this study and previously.[20] However, as shown here and also in a previous study, administration of Cy to NOD mice results in an initial abrupt decline in the severity of insulitis followed by a sharp and sustained increase until diabetes supervenes.[31] The reasons for the initial rapid decline in insulitis after Cy treatment are unclear. Mice fed nicotinamide or Prosobee or in combination showed significantly less insulitis and in particular, during the first 7 days after Cy treatment. Exposure of NOD mice to oral nicotinamide and maintained on the standard chow diet showed less insulitis at day 90 than in control mice.[3] However, the same degree of suppression was not observed when given Prosobee despite protection from disease.[5] The marked suppression of insulitis achieved in the present study with Prosobee plus nicotinamide is comparable to that achieved by the combined diet during spontaneous diabetes.[5] It is possible that the combination diet may contribute significantly to beta cell resistance and/or immune protection. The persistence of insulitis in diabetes-free mice observed in this study following dietary manipulation or by other interventions, such as oral insulin, suggests that a Th2-type cytokine milieu may predominate in the infiltrated islet.[32,33] This may also be true in the BB rat.[34]

Following Cy administration to female NOD mice, insulitis reaches its peak immediately before diabetes, at day 11 (20; this study). Thus, in this study, pancreatic tissues were analysed at day 11 to determine whether numerical differences in intra-islet CD4 and CD8 T-cell and macrophage phenotypes in the five experimental groups may contribute to protection or promotion of disease. In the three dietary groups, and in control mice not receiving Cy, the number of the three cell-types within the islets were significantly lower than in Cy-treated mice on the standard diet. This reduction closely mirrored the suppression of insulitis seen in the various groups. Thus, dietary prophylaxis may attenuate beta cell directed immune reactivity and preserve sufficient beta cell mass to maintain normoglycaemia. The protective diets, particularly, the soy protein may also be modulating the gut associated lymphoid system in favour of a Th2 cytokine bias.[35]

Cy given to NOD mice on the standard diet results in a marked up-regulation of iNOS in intra-islet macrophages, a major source of the enzyme.[20,21] The marked inhibition of intra-islet iNOS labelling in mice exposed to the three diabetes-preventing diets is noteworthy. Thus, production of NO, following transcription and translation of iNOS, may be a critical effector molecule mediating beta cell destruction in this model. The marked expression of iNOS is also seen in the later stages of Cy-induced diabetes.[20] However, in the present study, all three groups exposed to the anti-diabetogenic diets showed only a modest increase in intra-islet iNOS and at the later stages of Cy administration and despite significant macrophage infiltration in several islets. Therefore, nicotinamide and Prosobee may down-regulate iNOS expression and thus limit intra-islet NO production. Macrophages are thought to mediate beta cell destruction through NO production. Exposure of rat islets to activated macrophages in culture causes beta cell lysis which is prevented by co-incubation with nicotinamide.[36,37] A similar effect is seen following exposure of rat islets to nitrogen donors such as sodium nitroprusside.[38] Interleukin-1$\beta$ has been shown to promote gene expression of iNOS through activation of the transcription factor NFKB in rat islets or rat insulinoma cell line.[39] Purified rat islets when co-incubated with nicotinamide inhibits interleukin-1$\beta$-induced NO production in isolated rat islets and protects beta cells from destruction.[40] High nicotinamide concentrations, *i.e.*, 10 mM, or more reduce IL-1-induced NO formation from isolated islets and from RINm5F cells.[41] Under these conditions, nicotinamide may have post-transcriptionally regulated iNOS expression. However, at 50 mM, nicotinamide also reduced iNOS gene expression.[40] Nicotinamide and Prosobee may, therefore, inhibit iNOS gene expression and its translation *in vivo*. A similar inhibitory effect on iNOS gene expression is seen in lipopolysaccharide-activated macrophages following linomide therapy in MRL/Mp-*lpr*/*lpr* mice.[42] Interestingly, linomide also prevents diabetes in the NOD mouse.[43] Administration of anti-IL-1$\beta$ protects NOD mice from

Cy-induced diabetes.[44] Whether the protective diets employed in this study also down-regulate intra-islet interleukin-$\beta$ expression are unclear. Nicotinamide may also directly protect beta cells from immune attack by inhibiting beta cell poly (ADP) ribose polymerase (PARP) or promote beta cell regeneration.[45-49]

In conclusion, this study shows that nicotinamide or Prosobee, either given singly or in combination, successfully prevent the more aggressive form of diabetes in the NOD mouse induced by Cy. The three dietary treatments suppress insulitis and down-regulate the expression of intra-islet iNOS. These anti-diabetogenic effects may result in beta cell resistance and attenuation of beta cell directed cytotoxic processes. The present findings in the Cy model provide scientific rationale for further research aimed at implementing non-invasive dietary interventions for the prevention of IDDM in humans.

### Acknowledgments

Financial assistance from the Auckland Medical Research Foundation is gratefully acknowledged. We thank Ms. May Young for preparing the graphs and Professor R. B. Elliott for his support. We also thank Professor C. Nathan for advice regarding the specificity of the anti-iNOS used in this study.

### References

[1] Pozzilli, P. (1998). Prevention of insulin-dependent diabetes mellitus. *Diabetes/Metab. Rev.*, **14**, 69-84.

[2] Yamada, K., Nonaka, K., Hanafusa, T., Miyazaki, A., Toyoshima, H. and Tarui, S. (1982). Preventive and therapeutic effects of large-dose nicotinamide injections on diabetes associated with insulitis: an observation in nonobese diabetic (NOD) mice. *Diabetes*, **31**, 749-753.

[3] Reddy, S., Bibby, N. J. and Elliott, R. B. (1990). Early nicotinamide treatment in the NOD mouse: effects on diabetes and insulitis suppression and autoantibody levels. *Diabetes Res.*, **15**, 95-102.

[4] Elliott, R. B., Reddy, S. N., Bibby, N. J. and Kida, J. (1988). Dietary prevention of diabetes in the non-obese diabetic mouse. *Diabetologia*, **31**, 62-64.

[5] Reddy, S., Bibby, N. J., Wu, D., Swinney, C., Barrow, G. and Elliott, R. B. (1995). A combined casein-free-nicotinamide diet prevents diabetes in the NOD mouse with minimum insulitis. *Diabetes Res. Clin. Prac.*, **29**, 83-92.

[6] Dahl-Jorgensen, K., Joner, G. and Hanssen, K. F. (1991). Relationship between cow's milk consumption and incidence of IDDM in childhood. *Diabetes Care*, **14**, 1081-1083.

[7] Virtanen, S. M., Rasanen, L., Ylonen, K., Aro, A., Clayton, D., Langholz, B., Pitkaniemi, J., Savilahti, E., Lounamaa, R., Tuomilehto, J., Akerblom, H. K. and The Childhood in Diabetes in Finland Study Group (1993). Early introduction of dairy products associated with increased risk of IDDM in Finnish children. *Diabetes*, **42**, 1786-1790.

[8] Virtanen, S. M., Saukkonen, T., Savilahti, E., Ylonen, K., Rasanen, L., Aro, A., Knip, M., Tuomilehto, J., Akerblom, H. K. and the Childhood Diabetes in Finland Study Group (1994). Diet, cow's milk protein antibodies and the risk of IDDM in Finnish children. *Diabetologia*, **37**, 381-387.

[9] Karjalainen, J., Martin, J. M., Knip, M., Ilonen, J., Robinson, B. H., Savilahti, E., Akerblom, H. K. and Dosch, H.-M. (1992). A bovine albumin peptide as a possible trigger of insulin-dependent diabetes mellitus. *N. Eng. J. Med.*, **327**, 302-307.

[10] Cavallo, M. G., Fava, D., Monetini, L., Barone, F. and Pozzilli, P. (1996). Cell-mediated immune response to $\beta$ casein in recent-onset insulin-dependent diabetes: implications for disease pathogenesis. *Lancet*, **348**, 926-928.

[11] Scott, F. W., Norris, J. M. and Kolb, H. (1996). Milk and type 1 diabetes. *Diabetes Care*, **19**, 379-383.

[12] Atkinson, M. A., Bowman, M. A., Kao, K.-J., Campbell, L., Dush, P. J., Shah, S. C., Simell, O. and Maclaren, N. K. (1993). Lack of immune responsiveness to bovine serum albumin in insulin-dependent diabetes. *N. Eng. J. Med.*, **329**, 1853-1858.

[13] Paxson, J. A., Weber, J. G. and Kulczycki, A. Jr. (1997). Cow's milk-free diet does not prevent diabetes in NOD mice. *Diabetes*, **46**, 1711-1717.

[14] Harrison, L. C. and Honeyman, M. C. (1999). Cow's milk and type 1 diabetes: the real debate is about mucosal immune function. *Diabetes*, **48**, 1501-1507.

[15] Akerblom, H. K., Savilahti, E., Saukkonen, T. T., Paganus, A., Virtanen, S. M., Teramo, K., Knip, M., Ilonen, J., Reijonen, H., Karjalainen, J., Vaarala, O. and Reunanen, A. (1993). The case for elimination of cow's milk in early infancy in the prevention of type 1 diabetes: the Finnish experience. *Diabetes Metab. Rev.*, **9**, 269-278.

[16] Elliott, R. B. and Chase, H. P. (1991). Prevention or delay of type 1 (insulin-dependent) diabetes mellitus in children using nicotinamide. *Diabetologia*, **34**, 362-365.

[17] Elliott, R. B., Pilcher, C. C., Fergusson, D. M. and Stewart, A. W. (1996). A population based strategy to prevent insulin-dependent diabetes using nicotinamide. *J. Paediatric Endocrinol. Metab.*, **9**, 501-509.

[18] Lampeter, E. F., Klinghammer, A., Scherbaum, W. A., Heinze, E., Haastert, B., Giani, G. and Kolb, H. and the DENIS Group (1998). The Deutsche Nicotinamide

Intervention Study: an attempt to prevent type 1 diabetes. *Diabetes*, **47**, 980–984.
[19] Harada, M. and Makino, S. (1984). Promotion of spontaneous diabetes in non-obese diabetes-prone mice by cyclophosphamide. *Diabetologia*, **27**, 604–606.
[20] Reddy, S., Yip, S., Karanam, M., Poole, C. A. and Ross, J. M. (1999). An immunohistochemical study of macrophage influx and the co-localization of inducible nitric oxide synthase in the pancreas of non-obese diabetic (NOD) mice during disease acceleration with cyclophosphamide. *Histochem. J.*, **31**, 303–314.
[21] Rothe, H., Faust, A., Schade, U., Kleemann, R., Bosse, G., Hibino, T., Martin, S. and Kolb, H. (1994). Cyclophosphamide treatment of female non-obese diabetic mice causes enhanced expression of inducible nitric oxide synthase and interferon-gamma, but not of interleukin-4. *Diabetologia*, **37**, 1154–1158.
[22] Xie, Q.-W., Cho, H. J., Calaycay, J., Mumford, R. A., Swiderek, K. M., Lee, T. D., Ding, A., Troso, T. and Nathan, C. (1992). Cloning and characterization of inducible nitric oxide synthase from mouse macrophages. *Science*, **256**, 225–228.
[23] Kleemann, R., Rothe, H., Kolb-Bachofen, V., Xie, Q.-W., Nathan, C., Martin, S. and Kolb, H. (1993). Transcription and translation of inducible nitric oxide synthase in the pancreas of prediabetic BB rats. *FEBS Lett.*, **328**, 9–12.
[24] Reddy, S., Kaill, S., Poole, C. A. and Ross, J. (1977). Inducible nitric oxide synthase in pancreatic islets of the non-obese diabetic mouse: a light and confocal microscopical study of its ontogeny, co-localization and up-regulation following cytokine administration. *Histochem. J.*, **29**, 53–64.
[25] Reddy, S., Liu, W. and Elliott, R. B. (1993). Distribution of pancreatic macrophages preceding and during early insulitis in young NOD mice. *Pancreas*, **8**, 602–608.
[26] Reddy, S., Wu, D., Swinney, C. and Elliott, R. B. (1995). Immunohistochemical analyses of pancreatic macrophages and CD4 and CD8 *T*-cell subsets prior to and following diabetes in the NOD mouse. *Pancreas*, **11**, 16–25.
[27] Coleman, D. L., Kuzava, J. E. and Leiter, E. H. (1990). Effect of diet on incidence of diabetes in non-obese diabetic mice. *Diabetes*, **39**, 432–436.
[28] Karges, W., Hammond-McKibben, D., Cheung, R. K., Visconti, M., Shibuya, N., Kemp, D. and Dosch, H.-M. (1997). Immunological aspects of nutritional diabetes prevention in NOD mice: a pilot study for the cow's milk-based IDDM prevention trial. *Diabetes*, **46**, 557–564.
[29] Pontesilli, O., Carotenuto, P., Gazda, P. L., Pratt, P. F. and Prowse, S. J. (1987). Circulating lymphocyte populations and autoantibodies in non-obese diabetic (NOD) mice: a longitudinal study. *Clin. Exp. Immunol.*, **70**, 84–93.
[30] Reddy, S., Bibby, N. J. and Elliott, R. B. (1988). Ontogeny of islet-cell autoantibodies and insulitis in the non-obese diabetic mouse. *Diabetologia*, **31**, 322–328.
[31] Charlton, B., Bacelj, A., Slattery, R. M. and Mandel, T. E. (1989). Cyclophosphamide-induced diabetes in NOD/WEHI mice: evidence for suppression in spontaneous autoimmune diabetes mellitus. *Diabetes*, **38**, 441–447.
[32] Hancock, W. W., Polanski, M., Zhang, J., Blogg, N. and Weiner, H. L. (1995). Suppression of insulitis in non-obese diabetic (NOD) mice by oral insulin administration is associated with selective expression of interleukin-4, -10, transforming growth factor-$\beta$, and prostaglandin-E. *Am. J. Pathol.*, **147**, 1193–1199.
[33] Hermitte, L., Atlan-Gepner, C., Payan, M. J., Mehelleb, M. and Vialettes, B. (1995). Dietary protection against diabetes in NOD mice: lack of a major change in the immune system. *Diabetes Metab.*, **21**, 261–268.
[34] Scott, F. W., Cloutier, H. E., Kleemann, R., Woerz-Pagenstert, U., Rowsell, P., Modler, H. W. and Kolb, H. (1997). Potential mechanisms by which certain foods promote or inhibit the development of spontaneous diabetes in BB rats: dose, timing, early effect on islet area, and switch in infiltrate from Th1 to Th2 cells. *Diabetes*, **46**, 589–598.
[35] Weiner, H. L. (1996). Oral tolerance. In: *Prediction, Prevention and Genetic Counselling in IDDM*, Edited by Palmer, J. P., pp. 293–315, New York, John Wiley.
[36] Kolb, H., Burkart, V., Appels, B., Hanenberg, H., Kantwerk-Funke, G., Kiesel, U., Funda, J., Schraermeyer, U. and Kolb-Bachofen, V. (1990). Essential contribution of macrophages to islet cell destruction *in vivo* and *in vitro*. *J. Autoimmun.*, **3**, 117–120.
[37] Burkart, V. and Kolb, H. (1993). Protection of islet cells from inflammatory cell death *in vitro*. *Clin. Exp. Immunol.*, **93**, 273–278.
[38] Kallmann, B., Burkart, V., Kroncke, K.-D., Kolb-Bachofen, V. and Kolb, H. (1992). Toxicity of chemically generated nitric oxide towards pancreatic islet cells can be prevented by nicotinamide. *Life Sci.*, **51**, 671–678.
[39] Kwon, G., Corbett, J. A., Rodi, C. P., Sullivan, P. and McDaniel, M. L. (1995). Interleukin-1$\beta$-induced nitric oxide synthase expression by rat pancreatic $\beta$-cells: evidence for the involvement of nuclear factor $\kappa$B in the signalling mechanism. *Endocrinology*, **136**, 4790–4795.
[40] Andersen, H. U., Jorgensen, K. H., Egeberg, J., Mandrup-Poulsen, T. and Nerup, J. (1994). Nicotinamide prevents interleukin-1 effects on accumulated insulin release and nitric oxide production in rat islets of Langerhans. *Diabetes*, **43**, 770–777.
[41] Cetkovic-Cvrlje, M., Sandler, S. and Eizirik, D. L. (1993). Nicotinamide and dexamethasone inhibit interleukin-1-induced nitric oxide production by RINm5F cells without decreasing messenger ribonucleic acid expression for nitric oxide synthase. *Endocrinology*, **133**, 1739–1743.
[42] Hortelano, S., Diaz-Guerra, M. J. M., Gonzalez-Garcia, A., Leonardo, E., Gamallo, C., Bosca, L. and Martinez-A. C. (1997). Linomide administration to mice attenuates the induction of nitric oxide synthase elicited by lipopolysaccharide-activated macrophages and prevents nephritis in MRL/Mp-*lpr*/*lpr* mice. *J. Immunol.*, **158**, 1402–1408.
[43] Gross, D. J., Sidi, H., Weiss, L., Kalland, T., Rosenmann, E. and Slavin, S. (1994). Prevention of diabetes mellitus in non-obese diabetic mice by Linomide, a novel immunomodulating drug. *Diabetologia*, **37**, 1195–1201.
[44] Cailleau, C., Diu-Hercend, A., Ruuth, E., Westwood, R. and Carnaud, C. (1997). Treatment with neutralizing antibodies specific for IL-1$\beta$ prevents cyclophosphamide-induced diabetes in non-obese diabetic mice. *Diabetes*, **46**, 937–940.

[45] Sandler, S. and Andersson, A. (1986). Long-term effects of exposure of pancreatic islets to nicotinamide *in vitro* on DNA synthesis, metabolism and *B*-cell function. *Diabetologia*, **29**, 199–202.

[46] Sandler, S. and Andersson, A. (1986). Nicotinamide treatment stimulates cell replication in transplanted pancreatic islets. *Transplantation*, **46**, 30–31.

[47] Reddy, S., Salari-Lak, N. and Sandler, S. (1995). Long-term effects of nicotinamide-induced inhibition of poly(adenosine diphosphate-ribose) polymerase activity in rat pancreatic islets exposed to interleukin-1$\beta$. *Endocrinology*, **136**, 1907–1912.

[48] Pociot, F., Reimers, J. I. and Andersen, H. U. (1993). Nicotinamide-biological actions and therapeutic potential in diabetes prevention. *Diabetologia*, **36**, 574–576.

[49] Burkart, V., Wang, Z.-Q., Radons, J., Heller, B., Herceg, Z., Stingl, L., Wagner, E. F. and Kolb, H. (1999). Mice lacking the poly(ADP-ribose) polymerase gene are resistant to pancreatic beta-cell destruction and diabetes development induced by streptozotocin. *Nature Med.*, **5**, 314–319.

